# Improving the robustness of infant lexical processing speed measures

**DOI:** 10.3758/s13428-020-01385-5

**Published:** 2020-03-30

**Authors:** Julia Egger, Caroline F. Rowland, Christina Bergmann

**Affiliations:** 1 Max Planck Institute for Psycholinguistics, Wundtlaan 1, 6525XD Nijmegen, The Netherlands; 2 Donders Institute for Brain, Cognition and Behaviour, Radboud University, Nijmegen, The Netherlands

**Keywords:** Lexical speed of processing, Language development, Looking-while-listening paradigm, Eye-tracking

## Abstract

Visual reaction times to target pictures after naming events are an informative measurement in language acquisition research, because gaze shifts measured in looking-while-listening paradigms are an indicator of infants’ lexical speed of processing. This measure is very useful, as it can be applied from a young age onwards and has been linked to later language development. However, to obtain valid reaction times, the infant is required to switch the fixation of their eyes from a distractor to a target object. This means that usually at least half the trials have to be discarded—those where the participant is already fixating the target at the onset of the target word—so that no reaction time can be measured. With few trials, reliability suffers, which is especially problematic when studying individual differences. In order to solve this problem, we developed a gaze-triggered looking-while-listening paradigm. The trials do not differ from the original paradigm apart from the fact that the target object is chosen depending on the infant’s eye fixation before naming. The object the infant is looking at becomes the distractor and the other object is used as the target, requiring a fixation switch, and thus providing a reaction time. We tested our paradigm with forty-three 18-month-old infants, comparing the results to those from the original paradigm. The Gaze-triggered paradigm yielded more valid reaction time trials, as anticipated. The results of a ranked correlation between the conditions confirmed that the manipulated paradigm measures the same concept as the original paradigm.

## Introduction

Studying the language of children and infants is challenging. Even though infants and children comprehend utterances early on, taking measures that tell us what they understand can be difficult. To address this, Fernald and colleagues developed the looking-while-listening paradigm (Fernald, Pinto, Swingley, Weinbergy, & McRoberts, 1998; Fernald, Zangl, Portillo, & Marchman, 2008) based on a key insight from adult studies: that people tend to look at objects as they are labeled. In the looking-while-listening paradigm, participants are typically presented with two or more pictures of familiar objects at the same time and hear the label of one of the depicted objects. If they understand and recognize the label, participants will fixate on the labeled object (target) unconsciously and quickly. If they do so correctly significantly more often than we would expect by chance, we conclude that they comprehend the label. This way, children’s language comprehension can be measured online from a very early age onwards (the paradigm has been used successfully even in 6-month-olds; e.g., by Bergelson & Swingley, 2012). This paradigm has been vital in unraveling how infants begin comprehending words in real time (Fernald et al., 2008; Frank, Lewis, & MacDonald, 2016; Von Holzen & Bergmann, 2019).

However, the looking-while-listening paradigm can be used to do more than determine whether an infant understands a word: It can be used to study the dynamics of infant’s sentence processing, which can then inform theories of *how* and *why*, not just *when* infants acquire different linguistic skills. In particular, the speed with which young infants orientate their eyes to look at a familiar object in response to a label (e.g., *look at the dog*)—so-called lexical speed of processing—predicts new vocabulary growth. This finding has stimulated a number of suggestions about the relationship between familiar word processing and novel word learning. Specifically, Fernald and Marchman (2012) have shown a positive link between 18-month-old infants’ speed of processing and their productive vocabulary as reported by parents at 18, 21, 24 and 30 months (using the MacArthur-Bates CDI, Fenson et al., 2007); Fernald, Perfors, and Marchman (2006) have demonstrated that the speed with which 25-month-old infants process words was positively related to their productive vocabulary at 12, 18, and 21 months, and Marchman and Fernald (2008) have shown that children’s speed of processing at 25 months predicted working memory, IQ, and expressive vocabulary in the same children at 8 years of age.

These findings suggest an important link between how quickly infants can process familiar words and how easily they acquire new words. For example, Law and Edwards (2015: 19) have speculated that there is a causal link between processing speed and new word acquisition. They suggest that infants who can quickly recognize familiar words will, in consequence, be quicker to recognize unfamiliar words as novel, and thus will be able to more rapidly add new words to their vocabulary. Similarly, Fernald and colleagues have suggested that faster processing of familiar words frees up resources that can then be dedicated to the learning of new words (e.g., Fernald & Marchman, 2012). Beyond linking speech processing and later lexical development, Hurtado, Marchman, and Fernald (2008) have also reported a correlation between speed of processing and maternal speech input. For the first time, then, we have evidence that maternal input not only affects the trajectory of vocabulary acquisition, but also that it affects the speed with which infants process familiar words online. This, too, has important implications for our theories of acquisition, particularly those debating the role of the linguistic environment in infants' language learning. In sum, the ability to measure lexical speed of processing in the looking-while-listening paradigm has opened up new ways to think about the language acquisition process itself. Table 1 provides an overview of studies that have measured speed of processing and the findings it has engendered.

**Table 1:** An overview of studies measuring lexical speed of processing with participants in the second year of life

Study	*N* Participants	Age of participants (in months)	Number of trials	Mean number of RT trials	Range of RT Trials	Time window for RT analysis (in ms)	Mean RT (in ms)
Buckler, Oczak-Arsic, Siddiqui & Johnson (2017) - Experiment 1: Canadian accent condition	16	24	32	9.5	**-**	300–2300	657.85
Buckler, Oczak-Arsic, Siddiqui & Johnson (2017) - Experiment 1 Non-native accent condition	16	24	32	9.9	**-**	300–2300	774.48
Donnelly & Kidd (2020)	113	18	48*	14.2*	4–26*	300–1800	847.7*
Donnelly & Kidd (unpublished)	112*	21*	40*	13.63*	3–21*	300–1800	768.9*
Donnelly & Kidd (unpublished)	107*	24*	48*	11.91*	3–22*	300–1800	565.5*
Fernald & Hurtado (2006) - Experiment 1: Sentence frame	24	18	12	**-**	**-**	367–1800	906
Fernald & Hurtado (2006) Experiment 1: Word in isolation	24	18	12	**-**	**-**	367–1800	1034
Fernald & Hurtado (2006) Experiment 2: Sentence frame	24	18	12	**-**	**-**	367–1800	861
Fernald & Hurtado (2006) Experiment 2: Words with attention cue	24	18	12	**-**	**-**	367–1800	1015
Fernald & Marchman (2012) - Typically developing children	46	18	64	19.8	4–31	300–1800	789.1
Fernald & Marchman (2012) - Late talkers	36	18	64	18.9	3–32	300–1800	865.4
Fernald, Pinto, Swingley, Weinberg & McRoberts. (1998)	24	15	8	4.04*	-	200–2000*	995
Fernald, Pinto, Swingley, Weinberg & McRoberts. (1998)	24	18	8	4.91*	-	200–2000*	827
Fernald, Pinto, Swingley, Weinberg & McRoberts. (1998)	24	24	8	4.75*	-	200–2000*	679
Fernald, Marchman & Weisleder (2013) - High SES	47*	18	32	8.8*	2–16*	300–1800	746
Fernald, Marchman & Weisleder (2013) - Low SES	47*	18	32	8.8*	2–16*	300–1800	947
Fernald, Marchman & Weisleder (2013) - High SES	48	24	16	4.97*	2–10*	300–1800	666
Fernald, Marchman & Weisleder (2013) - Low SES	48	24	16	4.97*	2–10*	300–1800	802
Fernald, Perfors & Marchman (2006)	49*	15	24	5.77*	2–14*	300–1800	981
Fernald, Perfors & Marchman (2006)	44*	18	24	4.55*	2 – 9*	300–1800	962
Fernald, Perfors & Marchman (2006)	52*	21	24	6.48*	2–12*	300–1800	802
Fernald, Perfors & Marchman (2006)	57*	25	24	10.21*	2–17*	300–1800	771
Fernald, Swingley & Pinto (2001) - Experiment 1: Whole word condition	32	21	8	4.6* (across both age groups and conditions)	-	367–2000*	749.81
Fernald, Swingley & Pinto (2001) - Experiment 2: Whole word condition	32	18	8	4.6* (across both age groups and conditions)	-	367–2000*	943.31
Hurtado, Marchman & Fernald (2007)	18	18	16	6.3	2–13	367–1800	1084.9
Hurtado, Marchman & Fernald (2007)	15	24	16	6.3	2–13	367–1800	960 (estimate)
Hurtado, Marchman & Fernald (2007)	16	30	16	6.3	2–13	367–1800	851.8
Hurtado, Marchman & Fernald (2008)	27	18	32	8	2–18	300–1800	-
Hurtado, Marchman & Fernald (2008)	27	24	36	13	7–21	300–1800	-
Lany (2018) - Experiment 1	35	17	40	10	2–20	300–1800	839.8
Lany (2018) - Experiment 1	31	30	40	10	3–21	300–1800	617.9
Lany (2018) - Experiment 2	34	30	40	10	3–17	300–1800	671
Lany, Giglio & Oswald (2018a) - Easy words condition	45	12	16	2.76	2–6	300–1800	946.85
Lany, Giglio & Oswald (2018b) - Hard words condition	36	12	16	2.82	2–8	300–1800	957.92
Lany, Giglio & Oswald (2018a)	34	15–19	24	4.65	2–15	300–1800	910.82
Lany, Shoaib, Thompson & Estes (2018b) – Experiment 1	38	15–16	24	-	-	367–2200	1003.8
Lany, Shoaib, Thompson & Estes (2018b) – Experiment 2	30	15–15.9	24	-	-	367–2200	1052.7
Marchman et al. (2019) - Full term born children	63	18	64	19.8	2–32	300–1800	728
Marchman et al. (2019) - Preterm born children	69	18	64	15.7	2–33	300–1800	809
Peter et al. (2019)	80	19	64	11.95	2–27	300–1800	729.94
Peter et al. (2019)	73	25	60	10.41	2–24	300–1800	675.73
Peter et al. (2019)	74	31	64	10.48	2–24	300–1800	639.14
Swingley & Aslin (2000) - Correct pronunciation condition	56	18–23	12	7.26* (across both conditions)	-	367–2000*	718
Swingley & Aslin (2002) - Correct pronunciation condition	50*	15	24	5.86* (across both conditions)	-	367–2000*	922
Swingley & Fernald (2002) - Experiment 1	24	24	26* (including filler trials)	11.04* (across conditions)	-	367–2000*	808 (baseline trials)
Swingley & Fernald (2002) - Experiment 2	24*	24	28* (including filler trials)	10.79* (across conditions)	-	367–2000*	760 (baseline trials)
Swingley & Fernald (2002) - Experiment 3	24*	24	26* (including filler trials)	10.04* (across conditions)	-	367–2000*	-
Swingley, Pinto & Fernald (1999) - Experiment 1	32	24	16*	5.59*	-	200–2000*	785
Swingley, Pinto & Fernald (1999) - Experiment 2	32	24	16*	5.56*	-	200–2000*	746
Weisleder & Fernald (2013)	28*	19	32	9.21*	3–18*	300–1800	991.97*
Weisleder & Fernald (2013)	29	24	36	12.38*	4–18*	300–1800	814.74*
Zangl, Klarman, Thal, Fernald & Bates (2005) - Unaltered speech condition	95	12–31	24	45% of trials were distractor initial	-	625–2000	1144

Note: Information was extracted from the publications, unless marked with *, in which case the authors provided data directly

However, the looking-while-listening paradigm has one very important methodological limitation, which has serious consequences for its usefulness, and limits the reliability of the lexical speed of processing data collected. The visual reaction time data used to calculate the speed of processing measure requires that the infant shifts their fixation towards the target object upon hearing the object’s label. Thus, if their eyes are already fixated on the target object at the point of labeling on a particular trial, that trial cannot be included. In other words, we can only include trials in which the infant’s eyes are first fixated on the distractor, the second object on the screen, and then move towards the target object after it has been labeled. In addition, this shift must occur in a specific time window after naming, to allow us to make the inference that the shift is a consequence of the naming event (i.e., that it is causally linked to the naming event). When no gaze shift occurs, for example, because the infant is already fixating on the target object before naming, speed of processing cannot be calculated and the trial has to be discarded.

Since infants are, in principle, equally likely to fixate on either image before labeling, at least half the trials, but usually many more, are discarded in each experiment. Consequently, as shown in Table 1, most studies measure speed of processing based on only a few trials per infant (e.g., there were between 3 and 32 usable trials per infant, out of a total of 64 total trials reported, in Fernald & Marchman, 2012). Table 1 shows that much fewer than 50% of the trials can typically be used to calculate speed of processing.

With few trials, reliability suffers, for two reasons. First, calculating a measure from only a few trials per participant means that it is difficult to accurately estimate the true processing ability of any individual participant, which requires multiple observations. If a participant, for example, provides two reaction times, one very slow and another fast, the mean would be calculated and taken to index her individual speed of processing. However, from only two trials it is impossible to determine whether one of these should be seen as an outlier, or whether this average value between the two extremes indeed reflects the participant’s abilities accurately. Second, the paradigm often results in large variation in the number of usable trials for each participant, which means that we have a better estimate of the performance of participants with more trials, possibly skewing the results in a direction that deviates from the population, as fewer trials might lead to more extreme estimates. In addition, we currently have very little reliability data for speed of processing. Few previous studies measure speed of processing multiple times in the same children, and those that do have not reported correlations across time points (an exception is Peter et al., 2019, but their measures were taken 6 months apart). Speed of processing predicts vocabulary development, which allows conclusions about the validity of the measure, but not its reliability. In other words, if we measure the same infants twice, we do not know whether we would achieve similar results, particularly for those participants with only very few data points.

The issue of the reliability of estimating infants' speed of processing through visual reaction times has already received some consideration. For example, Fernald and Marchman (2012) have argued that more trials are important for an accurate measurement. They attributed their finding of a positive relationship between speed of processing and vocabulary growth at 18 months to the number of trials they secured per infant, in contrast to the results of Fernald et al. (2006), who did not find this effect. Fernald et al. obtained only a small number of trials per infant (range: 2–4), whereas Fernald and Marchman (2012) increased the number of trials per infant by introducing a second testing session. They concluded “[...] that meaningful individual differences in the efficiency of familiar word recognition are evident at ages younger than 2 years, if appropriate steps are taken to increase the stability and robustness of experimental measures of infants’ real-time interpretation of spoken language[...]” (p. 215). This example illustrates how securing more trials leads to a better estimate of the infant’s true capabilities. For those effects that have been shown repeatedly, most saliently the link with later lexical development, more reaction time trials, and thus more precise measures lead to more accurate effect size estimates. This, in turn, facilitates planning follow-up studies that aim to examine the cause of this relationship, for example, by allowing for sample size estimates that yield sufficient power.

The goal of the present paper was to introduce a manipulation to the classic looking-while-listening paradigm that selects the target based on the infant’s own gaze (Gaze-triggered). We anticipated that our manipulation would increase the number of usable visual reaction time trials without increasing the duration or number of test sessions, and thus yield more reliable estimates of individual infants' speed of processing. We tested Dutch infants at 18 months to facilitate comparison with data from previous studies, since this is an age group that has been frequently assessed on their speed of processing (see Table 1).

Infants took part in a looking-while-listening study with two conditions: one with our manipulated design (Gaze-triggered) design and one with the original design. To test our main objective, we ran two pre-registered analyses. First, we assessed whether the manipulation yielded more reaction time trials per infant than the original paradigm. Second, we correlated the reaction time data from the manipulated paradigm with the data from the original paradigm to determine whether the new design measured the same construct as the original design. We predicted that the correlation between reaction times in the two conditions would be high, suggesting that the two paradigms yield comparable individual differences rankings. In a final set of exploratory analyses, we (a) assessed correlations of infants' ranking within conditions to establish a baseline to compare against our between-condition correlation, since two separate tests cannot correlate more highly with each other than two instances of the same test; (b) tested whether there was an increase in reaction time over the course of the experiment in the novel paradigm to ensure that it did not have undesired effects on the speed of processing measure; (c) tested whether our conclusions hold both when taking into account all items tested, or only those that infants are reported to understand (see also Fernald et al., 2006), and (d) explored the relationship between speed of processing and the infants’ concurrent vocabulary size.

## Method

All materials we could freely share, depersonalized data, and analysis scripts are available on the Open Science Framework project website https://osf.io/8fwrb/.

### Participants

The main study included 43 Dutch-learning infants (mean age in days = 557.4, *SD* = 6.31, range: 548–570; 27 girls). Participants were recruited via a local babylab database of families who had signed up to take part in studies on child development. At the time of recruitment, we excluded infants who had a low birth weight (under 2500 g), any known visual or hearing impairments (including regular or recent prolonged ear infections), who were born prematurely (defined as 33 weeks of gestation or less), or whose parents had dyslexia. We also asked parents to estimate the amount of Dutch their infant heard regularly. We excluded infants who heard Dutch for fewer than six and a half days per week (equivalent to 93% Dutch input; this cut off allowed us to include only infants who are considered typically monolingual, in line with other infant language studies; Byers-Heinlein, 2015). We asked for parental education as a proxy of socio-economic status, in order to assess the homogeneity of our sample. On average, the parents of our participants had 17 years of formal education (range: 12–18 years), meaning that all of them obtained a qualification beyond high school level and the majority of them hold a university degree. The parents of one infant declined to answer this question.

Parents were contacted via phone or email and provided with information about the study. After agreeing to participate, they were invited to the lab and received several questionnaires by mail or e-mail to be filled in at home beforehand: the Dutch adaptation of the MacArthur Communicative Development Inventories (N-CDI; Zink & Lejaegere, 2002, adapted from Fenson et al., 1993), and lab-created questionnaires that contained questions about family background, daily activities, and home life (all these questionnaires are shared on the OSF project page). Scores on the questionnaires were not known to the experimenter at the point at which they tested participants.

Seventeen additional participants took part but were excluded after data collection for the following reasons: refusal to wear the target sticker needed for the eye-tracker (*N* = 3), technical failure (*N* = 3), fussiness (*N* = 1), visual impairment (*N* = 1), not fulfilling our monolingual input criterion after screening (*N* = 1), not providing enough valid trials for both experimental conditions (*N* = 6, see Analysis section below for details), having no trials where reaction time could be measured, or only providing reaction times in one condition (*N* = 2).

The study was first piloted with 13 participants in order to ensure that a within-subject-design would be feasible for 18-month-olds (i.e., we tested whether infants would complete a sufficient number of trials per condition to allow for analyses with sufficient power for our planned analyses; see below and pre-registration at https://osf.io/fqmuz/). The set-up was revised and improved before testing the main sample. None of the pilot participants were included in the final analyses.

### Materials

#### Visual stimuli

Stimuli were pictures of 16 different objects from four categories (food, animals, clothes, and toys). Four additional objects (cookie, spoon, baby, and bear) were chosen for the filler trials. We decided on our objects with the aim that all of them would be familiar to 18-month-old infants and easy to depict. For each object category, we used four different pictures of four different objects. The pictures appeared in yoked pairs, which we list in Table 2. The pairs were not matched in salience or frequency. Side of presentation was counterbalanced across trials.

**Table 2.** List of stimuli in their respective pairs

Item 1 (category) – Dutch translation	Item 2 (category) – Dutch translation
Apple (food) – Appel	Jacket (clothes) – Jas
Banana (food) – Banaan	Book (toys) – Boek
Bottle (food) – Fles	Ball (toys) – Bal
Bowl (food) – Kom	Shoe (clothes) – Schoen
Cat (animals) – Poes	(Woolen) Hat (clothes) – Muts
Cow (animals) – Koe	Sock (clothes) – Sok
Dog (animals) – Hond	Bike (toys) – Fiets
Horse (animals) – Paard	Car (toys) – Auto

As attention getters at the beginning of the trials, we picked six different animated videos with sound (from The ManyBabies Consortium, 2020; retrieved via https://osf.io/xbv95/). The calibration stimulus was the face of a cartoon character that moved to the five calibration points. This was used instead of a dot in order to engage the infants’ attention more effectively. The experiment started and ended with a child-friendly cartoon accompanied by instrumental music in order to draw the infants’ attention to the screen.

#### Auditory stimuli

A female native speaker of Dutch recorded the auditory stimuli in a sound-attenuated booth and was instructed to speak in a lively voice as if talking to an infant. Unlike previous studies, we did not present the target word in sentence context (Fernald et al., 1998; Fernald & Hurtado, 2006). Fernald and Hurtado (2006) have investigated the difference between presenting targets in sentence frames and in isolation, showing that while RTs might be slower, they still fall in the same distribution of RTs reported in the wider literature and are linked to identifying the correct target (see Table 1). This allowed for a more flexible onset of the target word in the Gaze-triggered paradigm. However, to remain as close as possible to the previous literature, we chose four exclamations that provided the context for our target words but that could be followed by a small pause in case the infant did not fixate on one of the objects immediately (see Procedure for details). We wanted the combination of the carrier sentence and the target noun phrase to sound natural to the infant, even if there was a longer break between these. The main goal of the paradigm was to have as many usable trials as possible, taking into account other limitations. Four variations per exclamation were chosen (“Kijk!”, “Wat is dat nou?”, “Wat leuk!”, “Zie je het?”; English translation: “Look!”, “What is this?”, “How nice!”, “Do you see it?”) and were recorded with various intonations. The speaker also recorded all object labels combined with the indefinite article several times (for example: “een poes”; English translation: “a cat”). We selected four variations per item for the experiment. Additionally, eight filler sentences were recorded (“Waar is de baby/koekje/lepel/beer?” and “Zie je de baby/koekje/lepel/beer?”; English translation: “Where is the baby/cookie/spoon/bear?” and “Do you see the baby/cookie/spoon/bear?”). Parents listened to masking music via headphones. The music consisted of songs mixed with voices speaking at the same time.

### Equipment

The study took place in an observation lab equipped with four cameras. The eye movements were recorded using the Eyelink Duo Portable recording at 1000 Hz. Participants saw the visual stimuli on a HP Laptop Elitebook 859 G3 Notebook with a 15.6-inch screen (resolution: 1600 x 900). The audio was presented at approximately 55 dB via two Genelec monitor speakers positioned on each side of the laptop. For creating as well as presenting the experiment, we used Presentation Version 20.0 Build 10.19.17. To be able to observe the participants' general state and record the session, we linked a Logitech webcam livestream to a second HP laptop. The parents wore noise-cancelling headphones (Sony WHCH700N) and listened to the masking music on an MP3 player (SanDisk Clip Sport Plus Player) that was set to a comfortable level.

### Procedure

**Fig. 1:**
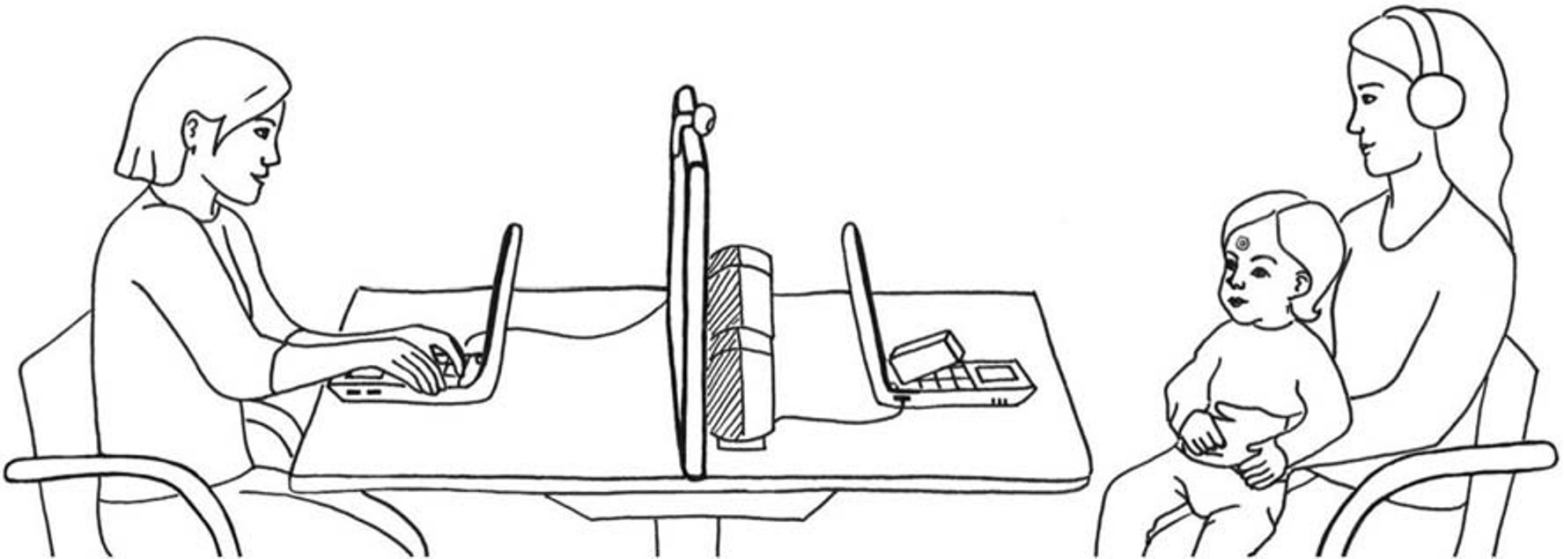
The experimental setup. The infant sat on their parent’s lap in front of the laptop with the eye tracker wearing a target sticker used by the eye tracker. The parent was listening to masking music via headphones. The experimenter sat on the other side of the table, not visible to the infant. They could control the experiment and view the infant via a webcam mounted on the partition. Reprinted from Methods, by N. Nota, 2019, Retrieved from 10.6084/m9.figshare.9976751.v1. Copyright 2019 by Naomi Nota. Reprinted with permission

The experiment took place in a darkened room. The infant sat on their parent’s lap approximately 50 cm away from the laptop screen. While the participant was watching a video with music, the experimenter placed a target sticker on the infant’s forehead, adjusted the eye tracker and arranged the headphones with masking music for the parents. The experimenter also started a recording of the session via a separate webcam. The experimental setup is depicted in Fig. 1.

**Fig. 2:**
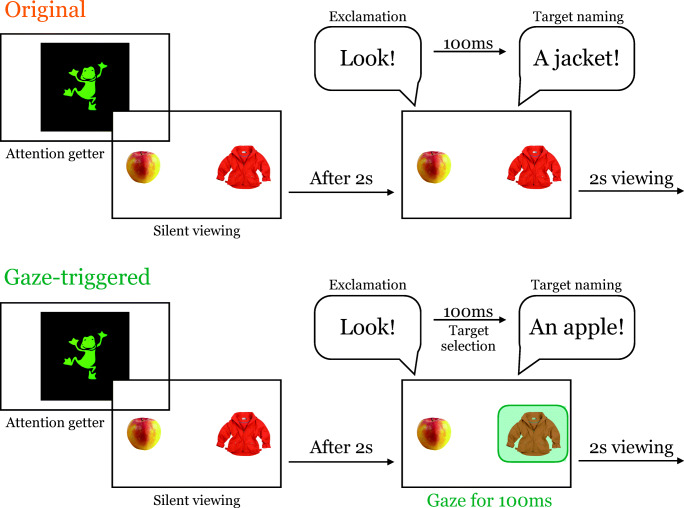
Illustration of a trial in both conditions. The Gaze-triggered condition does not differ visually from the original paradigm. The *blue area* represents the infant’s gaze triggering the naming event

After these preparations, participants completed a five-point calibration. Once calibration was successful, the first trial started. Figure 2 illustrates the course of a trial for both conditions. Each trial started with an attention getter, which was shown until the infant fixated on it for 500 ms or the experimenter pressed a button. Afterwards, two pictures appeared, one on the left and one on the right side of the screen. After 2 s of silent viewing, the infant heard one of the exclamations (see Materials). In the *Original* condition, one of the displayed items, the predetermined target, was named after 100 ms of silence. This condition models the standard looking-while-listening setup. In the *Gaze-triggered* condition, the target was chosen depending on the infant’s gaze. As soon as the infant looked at one of the two items for 100 ms in a set time window after the exclamation, this item became the distractor and the other item was named as target. The gaze of the infant was registered automatically by the eye tracker. In case the infant was not looking on the screen, the experimenter could trigger the onset of the target label by pressing a button to continue with the experiment. In both conditions, the trial continued for an additional 2 s after the onset of the target label. The average duration of a trial including the attention getter was 7 s.

The experiment consisted of 80 trials in total: 32 Gaze-triggered trials and 32 Original trials and 16 filler trials. The order of the conditions was mixed, alternating between blocks of eight Gaze-triggered trials and eight Original trials. Two filler trials were added to each eight-trial block, inserted pseudo-randomly between the first and eighth trial. This means that a filler trial was scripted to occur at any point, but never as the first or last trial of a block. Furthermore, there were never two consecutive filler trials. The condition that infants saw first was counterbalanced across participants. The experiment continued as long as the infant was attentive to the trials. If the participant failed to complete five trials in a row, the experimenter ended the session manually. At the end, regardless of whether the experiment was ended manually or the infant completed all 80 trials, the same video as shown at the beginning would play again. Participants completed on average 68 trials (range: 37–80), including filler trials.

We also introduced a feature that compensated for any bias in the Gaze-triggered condition that might be introduced if the infant always fixated on one of the objects (e.g., the apple in the pair in Fig. 2), which would mean that they always hear the other object (e.g., the jacket) labeled. We resolved this by deviating from the gaze-triggered approach if the infant fixated on the same object of a pair for the third time in a row; in this case the fixated object was labeled as a target. The same deviation occurred if the child fixated on the same object in the fourth trial in which the pair appeared. This manipulation meant that the infant heard the labels for all objects equally often. The experiment was programmed to keep track of the objects to control for this bias automatically. For each infant, a maximum of 16 trials could be affected by this bias correction (half of the Gaze-triggered trials). In our study, 11.5% on average of all possible trials were bias-corrected (range: 0–25%). We included trials with bias-correction as usable trials, but we could not compute speed of processing for these trials as the necessary shift in fixations did not occur. In theory, the infants were able to hear all items four times and see all pairs eight times throughout the experiment. This might have not been the case when the experiment had to be stopped earlier, because the infant has not been attentive to the trials five trials in a row.

The fact that the infants had to fixate on one of the items for at least 100 ms in the Gaze-triggered condition in order to elicit the target label meant that in some cases the delay between the onset of the exclamation and the onset of the target was longer in the Gaze-triggered condition (mean = 1285.32 ms, SD = 843.79, range: 710–7250 ms) than in the Original condition (mean = 961.91, SD = 171.52, range: 710–1220 ms) for trials analyzed here. This means participants saw the two images on average over 300 ms longer before onset of the label in the Gaze-triggered condition. We will address possible consequences of this in the Discussion section.

The eye-tracking session was followed by a 20-min play session in the same room (these data were used for a different study). These sessions were video recorded and are currently being transcribed and annotated. In an ongoing follow-up study, we are also tracing the language development of participants at 24, 30, and 36 months by inviting parents to fill in the N-CDI online. These data pertain to a different research question and we will not discuss them further in this article; they are mentioned for procedural completeness.

## Analysis

All analysis scripts can be found on the Open Science Framework project website https://osf.io/8fwrb/. Our analysis plan was pre-registered on the Open Science Framework after data collection was completed, but before any analyses were performed (https://osf.io/fqmuz/ on March 8, 2019). Additional analyses, including visual examination of the data, can be found on the project website. Deviating from our pre-registered plan, we decided to not report the analysis on accuracy (i.e., the proportion of fixations to the target after naming) here, given that our new paradigm changes the baseline considerably (from on average 50% pre-naming fixations on the target to near 0% fixations on the target).

We compared two conditions in this experiment, Gaze-triggered (i.e., dynamic selection of the target object based on infant gaze) and Original (i.e., the unchanged looking-while-listening design for measuring lexical speed of processing). The conditions did not differ from each other until the labeling of the target object took place (see Procedure for more details). We used a within-subject design with condition as the independent variable. We include number of valid trials, reaction time, trial number and target as dependent variables, depending on the analysis.

### Pre-processing

Before analysis, the raw eye-tracking data were transformed from edf-files to asc-files using the edf2asc translator program (documentation on http://download.sr-support.com/dispdoc/page25.html). These data were then pre-processed in R Version 3.5.0 (R Core Team, 2018) using RStudio Version 1.1.447 (RStudio Team, 2015) and the tidyverse package Version 1.2.1 (Wickham, 2017). Before further analysis, we removed the eye-movements recorded during calibration, filler trials, and attention getters. Additionally, we filtered the fixations assigned by the eye-tracker, such that we only included fixations that last for at least 100 ms in our analysis (cf. Casillas & Frank, 2017).

### Data analysis

For the analysis, all trials in which the infant looked at the screen for less than a total of 100 ms during the critical time window (0–2000 ms after target word onset) were excluded. This time span covers our reaction time window (300–1800 ms after target word onset). This yielded 21.02 (SD = 7.12) Gaze-triggered and 20.06 (SD = 7.46) Original potentially valid trials on average. The speed of processing (i.e., visual reaction time) measure was calculated only on trials where the infant looked at the distractor at the onset of the target label noun phrase. In order to be considered a valid visual reaction time, the shift in fixation from the distractor to the target had to occur between 300 and 1800 ms after the noun phrase onset. We chose the most commonly used time window based on the previous literature (see Table 1). Shifts that occurred earlier than 300 ms after onset were excluded, as infants are unlikely to be able to process the input and initiate the shift this quickly. Later shifts were excluded as these delayed shifts are most likely not a reaction to the target word. We only included participants who provided at least one trial with a valid reaction time for each condition. While in the literature most studies only include participants with at least two reaction time trials, we opted for having at least one trial, because the aim of our study is to compare how many reaction time trials we obtained, on average, in each condition, within participants.

The analyses were conducted in RStudio, using the following additional R packages: DescTools Version 0.99.28 (Signorell et al., 2019), dplyr Version 0.7.5 (Wickham, François, Henry, & Müller, 2018), lme4 Version 1.1–21 (Bates, Mächler, Bolker, & Walker, 2015), lmerTest Version 3.1-0 (Kuznetsova, Brockhoff, & Christensen, 2017), openxlsx Version 4.1.0 (Walker, 2018), reshape Version 0.8.8 (Wickham, 2007) and tidyr Version 0.8.1 (Wickham & Henry, 2018). For visualization, we used the package ggplot2 Version 3.1.0 (Wickham, 2016) and ggbeeswarm Version 0.2.3 (Clarke & Sherrill-Mix, 2017).

## Results

Our main objective was to increase the number of reaction time trials. Thus, the first analysis tested the prediction that the Gaze-triggered manipulation would yield more valid trials than the original paradigm. To quantify the number of valid trials and to account for the fact that infants completed different numbers of total trials or might have been distracted during the experiment, our dependent measure was the number of valid reaction time trials expressed as a percentage of total trials completed per condition. To obtain this percentage, we calculated the number of completed trials per condition for every participant as well as how many of these yielded a reaction time measure (i.e., yielded a shift from distractor to target within the pre-set time window; henceforth, valid trials). We then calculated the percentage of completed trials that yielded a valid reaction time measure for each condition.

**Fig. 3:**
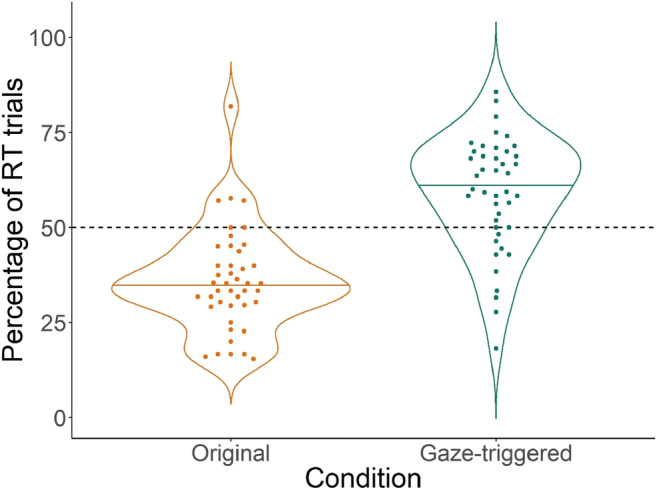
Violin plots of the percentage of valid reaction time trials per condition. The *dashed line* represents the 50% mark. Each *dot* indicates a participant per condition. The *colored lines* within the violins are the median across participants for each condition, while the *violin* outlines illustrate the distribution of participants

Figure 3 visualizes the mean percentage of valid trials per condition as well as the variance we observed. The Gaze-triggered condition yielded more valid trials than the Original condition (mean Gaze-triggered = 12.48, SD = 5.74, range: 4–25; mean Original = 7.2, SD = 3.7, range: 1–15). In comparison, past studies that have been administered similarly (32 trials at 18 months) have had a mean of eight to nine trials per participant (see Table 1, e.g., Fernald et al. 2013; Hurtado et al. 2008). We performed a one-sided paired *t* test with condition as the predictor variable and mean percentage of valid reaction time trials as the outcome variable. The test was one-sided because our prediction was directional in favor of the Gaze-triggered condition. We found a significant difference between conditions in the predicted direction (*t*(42) = 8.2, *p* < .001). As predicted, our manipulation increased the number of valid reaction time trials, yielding nearly twice as many valid trials on average as the original design.

The mean reaction time across participants was 929.54 ms (SD = 141.05, range: 658.38–1314.4 ms) in the Gaze-triggered condition and 948.5 ms (SD = 166.76, range: 672.91–1418 ms) in the Original condition. Our reaction times are in line with the literature for our age group (see Table 1, particularly e.g., Fernald et al., 2006; Fernald & Hurtado, 2006; who tested the same age group). There is no significant difference in the mean reaction times between the conditions (*t*(42) = – 0.69, *p* = 0.75). The lower standard deviation for reaction time in the Gaze-triggered condition compared to the standard deviation of the original paradigm can be seen as an indicator that the measures taken in the manipulated paradigm are less noisy, and are therefore more precise.

**Fig. 4:**
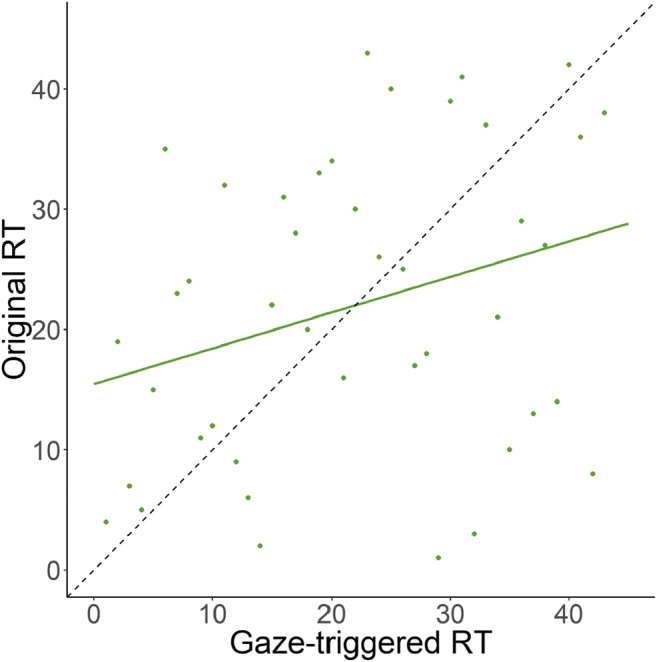
Scatterplot of the ranked average reaction time (RT) values for each participant between both conditions. The *dashed line* indicates what the ideal distribution of the data would be and the *colored line* represents the best fit to the data

Our second objective was to test whether the Gaze-triggered paradigm measures the same construct as the Original condition, by determining whether the individual ranks of speed of processing ability correlated between the two conditions. We decided to compare the ranks instead of the numeric values of the estimated reaction times, given that the conditions differ in the number of trials available to measure reaction times, which we expected to affect precision. Therefore, we computed the Spearman rank correlation coefficient between each participant's mean reaction time across conditions (Fig. 4). There was a significant, positive monotonic relationship between the scores in the two conditions (*rho* = .29, *n* = 43, *p* = 0.027, 95% CI [–0.004, 0.54]).

**Fig. 5:**
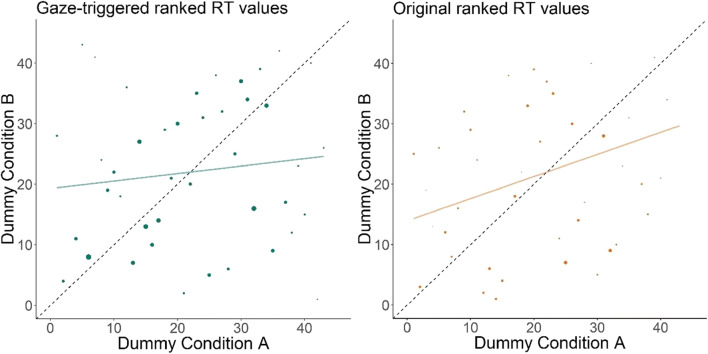
Scatterplot of dummy conditions created by subsetting reaction times (RTs) within participant within the Gaze-triggered (*left*) and the Original (*right*) condition. The size of the dots reflects the number of trials that were used for computing the mean reaction time per participant (range: 4–25 in the Gaze-triggered, 2–15 in the Original condition). The *dashed lines* indicate what the ideal distribution of the data would be and the *colored lines* represent the best fit to the data

The Spearman rank correlation coefficient between each participant's mean reaction time across conditions was significant but not large. However, since infant data tends to be noisy, it is difficult to judge whether this correlation is high enough to conclude that the two conditions are largely measuring the same construct. To aid our decision-making, we decided to assess the correlation within conditions in an exploratory analysis to provide a comparison score against which to judge the between-condition coefficient. We reasoned that a between-condition coefficient is unlikely to be much higher than the correlation coefficient yielded by comparing subsets of trials from the same condition. We randomly split the available trials per condition and per participant in half and assigned them to dummy conditions to compare visual reaction time values within participants and conditions. Note that power is necessarily lower in this analysis. Figure 5 presents scatterplots with the ranked reaction time values within condition (for the Original condition, we had to exclude two additional participants, as we had only one reaction time value available for these). For both the Gaze-triggered (*rho* = .12, 95% CI [–0.18, 0.4], *n* = 43, *p* = 0.43) and the Original (*rho* = .26, 95% CI [–0.04, 0.53], *n* = 41, *p* = 0.08) condition, the rank correlation was smaller than for the between-conditions analysis. Thus, we concluded that the Gaze-triggered manipulation is measuring the same construct as the original method; the speed with which individual infants are able to process lexical items.

At the same time, these values provide us with a test–retest reliability estimate for each condition, which is not available from previous studies, and provides an indicator of how accurate the estimate of visual reaction time is within participant. The fact that the correlation coefficients are small (below .3) indicate that even with the optimized Gaze-triggered design, there is a large amount of noise in the data, further underlining the need to obtain as many trials as possible per participant.

Initial feedback to the authors led to the concern that the infants might learn a pattern for the Gaze-triggered condition, given that they always have to shift their fixation after the onset of the target word. In the Gaze-triggered condition, it might be possible that the infants could learn, during the course of the experiment, that they would be required to shift their gaze from one object to another after hearing the exclamation uttered (e.g., “Kijk”). We thought this unlikely because the within-subjects design, plus the inclusion of the fillers, meant that under half the trials were gaze-triggered. However, to investigate this, we added a further exploratory analysis. We reasoned that if learning occurred, infants would become faster at reacting to the trials over the course of the experiment. Thus, we added a linear mixed effects regression model over the reaction times within the Gaze-triggered condition, to test if reaction time decreased with increasing trial number. We used trial number as a fixed effect and we included participant, target object, and target by participant as random factors.

$$ \mathrm{RT}\sim \mathrm{trial}+\left(1\ |\ \mathrm{Participant}\right)+\left(1\ |\ \mathrm{target}\right)+\left(1\ |\ \mathrm{target}/\mathrm{Participant}\right) $$

**Fig. 6:**
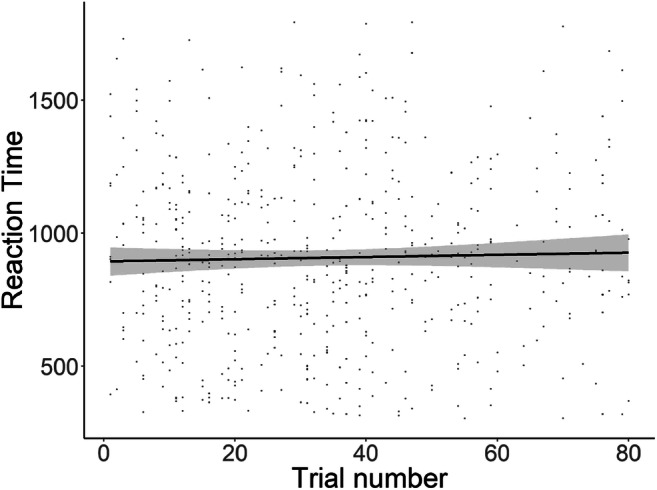
Scatterplot of Gaze-triggered reaction times across trial numbers. Each *dot* indicates the reaction time of a participant during a given trial. Towards the end of the *x*-axis, there are fewer dots as not all participants completed all 80 trials. Note that the order of conditions (Gaze-triggered and Original) were counterbalanced. The *black line* is the regression line and the *grey area* resembles the standard error

Table 3 shows the results. There was no effect of increasing trial number on the reaction times of the participants. Figure 6 further illustrates this finding.

**Table 3:** Linear mixed effects model on the RTs in the Gaze-triggered condition over the course of the experiment

	Estimate	Std. Error	*df*	*t* value	Pr (>|t|)
(Intercept)	865.088	41.483	42.403	20.854	<.001
trial	1.174	0.722	499.086	1.626	0.104

Additionally, since we collected CDI data from our participants at the time of testing, we were able to see if the infants comprehended the words we used in our experiment, according to their parents (see also Fernald et al. 2006). On average, 2.27 words (range: 0–11) of our items were reported as not comprehended by the parents. Thus, we re-ran the above analyses excluding the trials that contained the words that were unfamiliar to each participant, according to their parents. The results and conclusions do not differ substantially from those reported here, so we do not report further on these here (the plots and further reports on these analyses can be found in the supplemental materials on our project page on OSF). We also conducted a linear mixed effects regression model over the reaction times, to see whether the infants differed in their reaction times when a word was unfamiliar. We used whether the word was familiar as fixed effect (WordKnown) and added participant, target item, and target item by participant as random factors.

$$ \mathrm{RT}\sim \mathrm{WordKnown}+\left(1\ |\ \mathrm{Participant}\right)+\left(1\ |\ \mathrm{target}\right)+\left(1\ |\ \mathrm{target}/\mathrm{Participant}\right) $$

There was no significant change in the reaction times depending on the receptive familiarity of the word, as can be seen in Table 4.

**Table 4:** Linear mixed effects model on the RTs with word knowledge as fixed effect

	Estimate	Std. Error	*df*	*t* value	Pr (>|t|)
(Intercept)	974.11	45.33	94.99	21.491	<.001
WordKnownTRUE	– 63.15	41.09	344.48	– 1.537	0.125

Overall, the CDI scores of our infants reveal that they comprehend an average of 279.63 words (SD = 137.92, range: 48–684) and were able to produce an average of 57.35 words (SD = 48.18, range: 7–271). The individual scores can be found on the OSF project website (https://osf.io/8fwrb/).

Finally, we also explored the relationship between speed of processing and concurrent vocabulary size. This link has been frequently tested in the previous literature. We opted for a Spearman rank correlation because we wanted to investigate the link between processing speed and lexicon size without making strong assumptions regarding the exact numerical relationship between RTs (in milliseconds) and vocabulary (as measured by words produced according to parental report). For this analysis, we took the mean RTs of our infants across conditions, so that we would have at least two reaction times per infant. Following the literature, we used the expressive CDI score as measure for concurrent vocabulary size. There was a negative relationship between the rank of the RTs and the expressive vocabulary size that was significant at 0.06, though not at 0.05 (*rho* = –.24, *n* = 43, *p* = 0.054, 95% CI [– 0.51, 0.05]). The effect size is within the range reported in the previous literature.

## Discussion

The aim of this study was to improve the robustness of the looking-while-listening paradigm regarding the measurement of infants’ speed of processing. Our first objective was to increase the number of speed of processing trials with our manipulated, Gaze-triggered paradigm. Therefore, we compared the percentage of usable reaction time trials per participant between conditions. Our results showed that the Gaze-triggered paradigm yielded a significantly higher percentage of valid reaction time trials than the original paradigm. Because we increased the overall number of trials used to compute an estimate of participants' speed of processing, we conclude that the new paradigm allows us to obtain a more reliable estimate of their underlying abilities. Moreover, given that we observed a smaller range and standard deviation (i.e., less extreme values) in the Gaze-triggered condition, we conclude that the Gaze-triggered condition measures speed of processing more precisely and with less noise. Overall, our mean RTs of both conditions fall within the range of RTs reported in the literature (see Table 1, particularly studies testing the same age group: Fernald & Hurtado, 2006; Fernald et al., 2006; Fernald et al., 2001; Hurtado et al., 2007; Weisleder & Fernald, 2013).

Second, we predicted that our new paradigm would measure the same construct as the original paradigm. We tested this by correlating the individual ranks of the participants’ reaction time across conditions, and were able to demonstrate that the individual capabilities of the infants were comparable across conditions. We also conducted rank correlations within the conditions, which were smaller than the correlation between conditions. This supports our hypothesis that both the Gaze-triggered and the original paradigm measure the same construct. However, the fact that the correlations were not large, both within and across conditions shows that, while the measure of speed of processing has been widely used, it is prone to noise and might lead to conflicting results, as indicated by Fernald et al. (2006) and Fernald and Marchman (2012).

Additional exploratory analyses ruled out other interpretations. First, exploratory linear mixed effects model revealed that infants do not get faster over time in the Gaze-triggered condition. Thus, it is unlikely that the infants learnt, during the course of the experiment, that they would be required to shift their gaze from one object to another. Nevertheless, we would recommend including a substantial number of non-gaze-triggered trials (Fillers) when using the Gaze-triggered paradigm in order to disrupt any potential learning over the course of the experiment. Second, we explored post hoc if the reaction times differed when we excluded words that children did not know, according the parental reports using the N-CDI. Similarly to results reported by Fernald et al. (2006), we did not find an effect of word knowledge on the reaction times. Third, we investigated the correlation between speed of processing and concurrent expressive vocabulary score. Our results showed a marginally significant relationship, comparable with results in the previous literature (e.g., Fernald et al. 2013).

We also noted that because infants had to fixate on the target picture for at least 100 ms to hear the label, there was a resulting difference of about 300 ms in the duration of the pre-naming phase between conditions. Could this have affected our results? Indeed, infants might during this time become more familiar with the two images, possibly decreasing their reaction time. However, we do not observe a significant difference of reaction times between conditions and a correlation between ranks of reaction times within participants. Both results point to this difference not substantially altering our results, but further investigation is necessary to explore this issue.

In summary, we have shown that with a small manipulation of the original looking-while-listening paradigm, we can improve the speed of processing measure taken from infants.

### Future directions

With the new paradigm, we can measure lexical speed of processing more accurately and more robustly in future studies. The importance of this, especially in light of individual differences research, was already noted by Fernald and Marchman (2012). Past research has shown that lexical speed of processing predicts concurrent and future vocabulary size as measured by the CDI (e.g., Fernald et al., 2013) as well as aspects of maternal speech input (e.g., Hurtado et al., 2008). Are infants faster in processing familiar words due to their vocabulary knowledge or does their vocabulary grow faster due to their processing capabilities? With more trials and a more precise measure, it will be possible to address these questions, particularly using training or intervention designs to begin tapping into directional and causal relationships.

With more trials, it will also be possible to investigate the impact of item-level characteristics, such as frequency, semantic salience, priming, or phonological transparency on speed of processing. From the literature on adult language processing, we know that different features affect lexical processing as well as acquisition (e.g., Schilling, Rayner, & Chumbley, 1998; Sperber, McCauley, Ragain, & Weil, 1979). We can now extend this to early first language acquisition, because the new paradigm allows for item level analyses. The Gaze-triggered paradigm thus opens up new paths of research possibilities.

## Conclusions

This paper introduced a manipulated looking-while-listening paradigm to enhance the power of infants’ speed of processing measures by drastically increasing the number of reaction time trials per infant. The new Gaze-triggered paradigm is shown to measure the same construct as the original, but with a less noisy measure with increased power. With more trials, this new paradigm allows for more, and new, research opportunities.

### Open Practices Statement

The depersonalized data and all the materials we could share are available at https://osf.io/8fwrb/. The analysis plan was pre-registered (https://osf.io/fqmuz/) on March 8, 2019.
